# Goal-Oriented Respiratory Management for Critically Ill Patients with Acute Respiratory Distress Syndrome

**DOI:** 10.1155/2012/952168

**Published:** 2012-08-23

**Authors:** Carmen Sílvia Valente Barbas, Gustavo Faissol Janot Matos, Marcelo Britto Passos Amato, Carlos Roberto Ribeiro Carvalho

**Affiliations:** ^1^Respiratory ICU and LIM-09, Medical School, University of São Paulo, 6 Andar, Avenida Dr. Eneas de Carvalho Aguiar 155, 05403-000 São Paulo, SP, Brazil; ^2^Adult ICU Albert Einstein Hospital, 5 Andar, Avenida Albert Einstein 627, 05652-900 São Paulo, SP, Brazil

## Abstract

This paper, based on relevant literature articles and the authors' clinical experience, presents a goal-oriented respiratory management for critically ill patients with acute respiratory distress syndrome (ARDS) that can help improve clinicians' ability to care for these patients. Early recognition of ARDS modified risk factors and avoidance of aggravating factors during hospital stay such as nonprotective mechanical ventilation, multiple blood products transfusions, positive fluid balance, ventilator-associated pneumonia, and gastric aspiration can help decrease its incidence. An early extensive clinical, laboratory, and imaging evaluation of “at risk patients” allows a correct diagnosis of ARDS, assessment of comorbidities, and calculation of prognostic indices, so that a careful treatment can be planned. Rapid administration of antibiotics and resuscitative measures in case of sepsis and septic shock associated with protective ventilatory strategies and early short-term paralysis associated with differential ventilatory techniques (recruitment maneuvers with adequate positive end-expiratory pressure titration, prone position, and new extracorporeal membrane oxygenation techniques) in severe ARDS can help improve its prognosis. Revaluation of ARDS patients on the third day of evolution (Sequential Organ Failure Assessment (SOFA), biomarkers and response to infection therapy) allows changes in the initial treatment plans and can help decrease ARDS mortality.

## 1. Introduction

Acute respiratory distress syndrome (ARDS) is due to an increase in the pulmonary alveolar-capillary membrane permeability causing lung edema rich in protein and consequently acute hypoxemic respiratory failure in genetically susceptible patients exposed to determined risk factors [1–14]. A recent study showed that the del/del genotype (patients homozygous for the 4 base pair deletion in the promoter of NFKB1) is associated with an age-dependent increase in odds of developing ARDS (OR 5.21, 95% CI 1.35–20.0) and patients with the del/del genotype and ARDS also have increased hazard of 60-day mortality (HR 1.54, 95% CI 1.01–2.36) and more organ failure (*P* < 0.001) [15]. All age groups may be affected, although the syndrome has a higher incidence and mortality in older people [16]. The most common precipitating causes of ARDS are pulmonary infections, nonpulmonary sepsis, shock, gastric aspiration, thoracic trauma, fat embolism, near drowning, inhalational injury, cardiopulmonary bypass, drug overdose, acute pancreatitis, and high-risk trauma (especially traumatic brain injury) [17]. Recent epidemiological studies suggested a variety of intrahospital risk factors for ARDS development such as multiple blood products transfusions, mechanical ventilation with high tidal volumes, excessive fluid resuscitation, and hospital-acquired pneumonia as well as high-risk surgeries (especially aortic vascular, cardiac, and acute abdomen); all risk factors are potentially preventable. Chronic alcohol abuse, chronic liver disease, immunosuppression, hypoalbuminemia, and obesity are also all associated with the development of ARDS, whereas diabetes mellitus appears to be protective [17].

After exposure to a risk factor, there is an important activation of neutrophils and release of harmful mediators including cytokines (such as interleukins 1, 6, and 8 and soluble tumor necrosis factor-alpha receptors), proteases, reactive oxygen species, and matrix metalloproteinases leading to future damage. An overwhelming pulmonary inflammatory process is initiated leading to alveolar epithelial and vascular endothelial injury. Alveolar epithelial injury of type I cells contributes to the pulmonary edema and the breakdown of this epithelial barrier exposes the underlying basement membrane, predisposing to bacteremia and sepsis. Injury to type II alveolar cells leads to an impairment of surfactant function with consequent collapse of the lungs. Histopathologically there is diffuse alveolar damage with neutrophil infiltration, alveolar hemorrhage and hyaline membrane formation [18–22]. There are localized destruction and occlusion of the vascular bed of the lungs by intravascular thrombosis and an increment of the anatomical dead space resulting in an increase of arterial carbon dioxide associated with a poor outcome. Fibrosis can be evident histologically as early as one week after the onset of ARDS and procollagen III peptide, a precursor of collagen synthesis, can be elevated in bronchoalveolar lavage fluid of ARDS patients at the time of tracheal intubation, its increment being associated with a poor ARDS prognosis. Vascular injury and remodeling may lead to pulmonary arterial hypertension which may compromise right ventricular function associated with a poor clinical outcome [13].

## 2. Importance of Early Recognition of ARDS and Its Correct Diagnosis

Incorporation of modified risk factors such as acute increase of respiratory rate, presence of tachypnea, detection of pulse oximeter desaturation, increased necessity of oxygen supplementation, presence of low pH, acidosis, or hypoxemia in an arterial blood gas sample in clinical practice can improve the clinicians' ability to perform early diagnosis and prompt therapeutic intervention in ARDS [17]. The presence of these modified risk factors may alert physicians to avoid secondary hospital exposures, such as blood products transfusions, excessive fluid administration, infusion of potentially toxic drugs, high tidal volume mechanical ventilation, and gastric aspiration.

Implementation of ventilator associated pneumonia prevention bundles decreases the incidence of VAP and can lower the incidence of ARDS [17]. Implementation of automated ARDS electronic screening in USA hospitals such as “ASSIST” (electronic alert from laboratory when the arterial blood gas analysis shows hypoxemia and the radiology department when chest X-ray shows bilateral pulmonary infiltrates) to identify intubated patients with ARDS in medical and surgical ICUs showed a sensitivity of 97.6% (95% CI, 96.8–98.4%) and specificity of 96.8% (95% CI, 96.8–98.4%) when compared to a manual screening algorithm that had a sensitivity of 57.1% (95% CI, 54.5–59.8%) and specificity of 99.7% (95% CI, 99.4–100%) in 1270 ICU patients over a 21-week period during enrollment in ARDSNet trials [23]. The results of this study indicated the advantages of having an in-hospital automated screening of ARDS over manual screening. The automated screening can increase the chances of ARDS diagnosis, alert the clinicians, and elicit the rapid response from the hospital team of intensivists to initiate clinical protocols and ARDS therapeutic interventions [24].

Most hospitals and intensive care units worldwide use the standard criteria for the diagnosis of acute lung injury (ALI)/ARDS: presence of acute hypoxemia (PaO_2_/FIO_2_ less than 300 mmHg or 39.99 Kpa for ALI or less than 200 mmHg or 26.66 Kpa for ARDS), bilateral infiltrates seen on a frontal chest radiograph that are consistent with pulmonary edema, and no clinical evidence of left atrial hypertension, or (if it is measured) a pulmonary artery wedge pressure (PAWP) of less than 18 mmHg according to the 1994–1998 American-European Consensus Conference on ARDS (AECC) [25, 26]. This definition aimed to simplify and standardize the diagnosis of ARDS worldwide. However, in clinical practice, in order to detect and diagnose ALI/ARDS cases, physicians must focus on patients' complaints, physical examination alterations, patients at risk of developing the disease, or patients presenting finger pulse oximeter desaturation. Following the ALI/ARDS clinical suspicion, physicians should order an arterial blood gas analysis and a chest radiograph to be able to confirm the ALI/ARDS diagnosis. Recent updates of ARDS definition such as the 2005 Delphi consensus [8] or the Berlin definition [27] were published in order to improve ARDS diagnosis criteria. The Berlin definition reclassified ARDS as mild (PaO_2_/FIO_2_ < 300 or 39.99 Kpa), moderate (PaO_2_/FIO_2_ < 200 or 26.66 Kpa), and severe (PaO_2_/FIO_2_ < 100 mmHg or 13.33 Kpa) and removed the term ALI and the necessity of a Swan Ganz catheter to access PAWP. Acute time frame was specified as the onset within 1 week of a known clinical insult or new or worsening respiratory symptoms chest radiography criteria were clarified and bilateral opacities consistent with pulmonary edema were maintained as the main radiological criteria of ARDS, but it was recognized that these findings could be demonstrated on CT scan instead of chest radiograph. The recent Berlin definition of ARDS is a decisive step forward in refining the diagnosis of the syndrome, but PaO_2_/FIO_2_ is influenced by ventilator settings and this fact should be considered; bilateral pulmonary infiltrates can be the result of a wide variety of acute lung diseases that should be better investigated. Left and right ventricular function, pulmonary artery pressures, and volemic status could be better evaluated by bedside echocardiography and extravascular lung water can be measured using PiCCO catheter, in order to evaluate the degree of pulmonary edema. Predictors of mortality should be calculated at ICU admission. With the information, the ICU team can program a more careful treatment plan according to disease severity. The Berlin definition shows better predictive validity for mortality compared to the AECC definition, but the absolute value of the area under the receiver operating curve is still too small (0.577), suggesting that some factors are still missing. Further discussion and research are needed before we reach a comprehensive definition of ARDS.

## 3. Importance of Computer Tomography to the Diagnosis of ARDS, to Access the Severity of the Disease, to Make Differential Diagnoses, and to Set the ARDS Ventilatory Strategy

The typical findings of ARDS in a computer tomography reveal a heterogeneous bilateral pulmonary infiltrate predominantly in gravity-dependent regions of the lungs and more preserved lungs in nondependent lung regions. Using quantitative analysis of the CT scan, the gravity-dependent pulmonary ARDS infiltrate is typically nonaerated lung tissue consistent with compressive atelectasis [28, 29]. Lung weight assessed by CT scan is increased in ARDS and is correlated with the severity of the syndrome [27].

The finding of concomitant interstitial infiltrates suggests viral or mycoplasma, chlamydia or opportunistic pulmonary infections, or drug-induced lung disease.

The differential diagnosis of bilateral pneumonia, alveolar hemorrhage, and acute interstitial lung disease such as acute interstitial pneumonia, hypersensitivity pneumonitis, acute eosinophilic pneumonia, and bronchiolitis obliterans with organizing pneumonia can be suggested by the characteristic CT scan findings of each specific disease [12].

**Figure 1:**
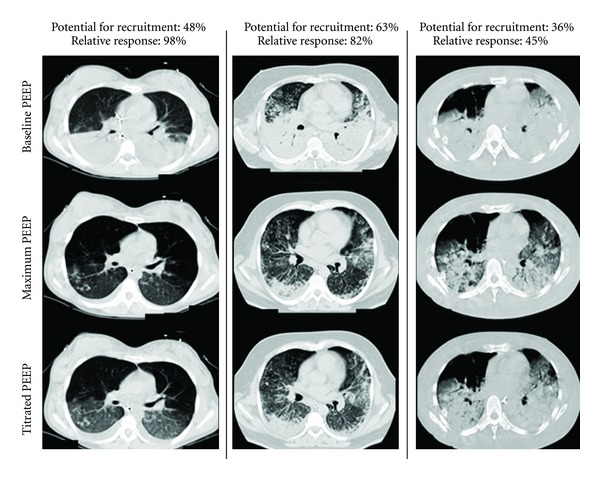
Computer tomographic evaluation of maximal recruitment strategy and adequate PEEP titration in early severe ARDS patients.

The results of stepwise lung recruitment maneuvers as well as positive end-expiratory (PEEP) titration to keep the lungs open with minimal collapse can be assessed by computer tomography analysis [30]. This strategy is aimed at opening up the lungs and keeping the lungs open [31] as quickly and early as possible as postulated by Lachmann [32] in order to have a huge improvement in lung function and avoid potential ventilator-induced lung injury. Recently, our group reported the experience with Maximal Recruitment Strategy (MRS) in 51 patients with ARDS. MRS consisted of 2-minute steps of tidal ventilation with pressure-controlled ventilation, fixed driving pressure of 15 cmH_2_O, respiratory rate of 10 breaths/minute, inspiratory/expiratory ratio of 1 : 1, and stepwise increments in PEEP levels from 10 to 45 cmH_2_O (recruitment phase). After that, PEEP was decreased to 25 cmH_2_O and, then, from 25 to 10 cmH_2_O (PEEP titration phase) in steps of 5 cmH_2_O, each one lasting 4 minutes. At each of the steps computer tomography image sequences from the carina to the diaphragm were acquired during an expiratory pause of 6–10 seconds. Lung collapse was assessed online by visual inspection, for immediate clinical decision, and offline for quantitative measurements. MRS showed a statistically significant decrease in nonaerated areas of the ARDS lungs that was accompanied by a significant increment in oxygenation. The opening plateau pressure observed during the recruitment protocol was 59.6 (±5.9 cmH_2_O), and the mean PEEP titrated after MRS was 24.6 (±2.9 cmH_2_O). Mean PaO_2_/FiO_2_ ratio increased from 125 (±43) to 300 (±103; *P* < 0.0001) after MRS and was sustained above 300 throughout seven days. Nonaerated parenchyma decreased significantly from 53.6% (interquartile range (IQR): 42.5 to 62.4) to 12.7% (IQR: 4.9 to 24.2) (*P* < 0.0001) after MRS. The potentially recruitable lung was estimated at 45% (IQR: 25 to 53), (Figure 1). ICU mortality was 28% and hospital mortality was 32%. The independent risk factors associated with mortality were older age and higher driving pressures (or higher delta pressure control). There were no significant clinical complications with MRS or barotrauma [33]. A better evolution of these ARDS patients with less necessity of oxygen supplementation in the recovery phase of the disease and a better quality of life must be tested in prospective, controlled clinical trials. A recent meta-analysis showing beneficial effects on mortality using higher PEEP levels compared with lower PEEP in ARDS patients corroborates the results of our clinical case series of ARDS patients submitted to MRS [33].

ARDS is a biphasic disease that progresses from an acute exudative phase, characterized by epithelial and endothelial injury, neutrophilic aggregation, formation of hyaline membranes, alveolar edema, and hemorrhage, to an organizing phase, characterized by regeneration and healing via resolution or repair with persistent intra-alveolar and interstitial fibrosis [11]. It is crucial to make the diagnosis of ARDS in the acute phase (preferably less than 72 hours) in order to make it possible to open up the lungs with recruitment maneuvers and keep the lungs open with sufficient PEEP levels to enable a more homogenous ventilation, minimizing the possible ventilator-induced lung injury (VILI) triggers and allowing the recovery of the lungs [34–36]. A recent study analyzing 85 patients with ARDS graded into six findings according to the extent of fibroproliferation at the CT scan showed that higher CT scores were associated with statistically significant decreases in organ-failure free days as well as ventilator free days and were an independent risk factor for mortality (OR = 1.2, 95% CI 1.06–1.36, *P* < 0.005) [37].

## 4. Other Imaging Techniques to Evaluate the ARDS Lungs

Positron emission tomography with (^18^F) fluorodeoxyglucose (FDG-PET) detects inflammatory cells and can assess lung inflammation in ARDS lungs helping in the understanding of ARDS physiopathology [38–40].

Lung ultrasonography is a new helpful tool that can be performed at bedside without radiation exposure. Thoracic ultrasound is widely used for diagnostic and therapeutic intervention in patients with pleural effusion and pneumothoraces. The assessment of lung recruitment and PEEP titration in ARDS patients at bedside using lung ultrasonography is a new promising technique [41]. Currently, the two main limitations of this technique are its inability to detect lung overdistension and its operator-dependent characteristic.

Thoracic electrical impedance tomography (EIT) is a highly promising imaging technique to apply at the bedside for PEEP titration in ARDS patients. New automated tools permit the calculation of the percentage of collapsed as well as overdistended lung tissue at decremental PEEP levels after lung recruitment maneuvers (Figure 3). The regional distribution of collapse and overdistension may provide insights about the lung pathology. This technique permits daily PEEP adjustments at the bedside and verification of tidal volume distribution, avoiding excessive end-expiratory collapse or tidal overdistention [42–45]. One of the main advantages of this technique is the possibility of around the clock monitoring. Further studies are needed to evaluate the clinical impact of these bedside techniques in ARDS patients' prognosis.

## 5. Importance of Noninvasive Ventilation in ARDS

Randomized trials suggested that patients with acute hypoxemic respiratory failure are less likely to require endotracheal intubation when noninvasive ventilation (NIV) is added to standard therapy [46]. However, most of these studies analyzed mixed causes of acute hypoxemic respiratory failure and reported the highest intubation rates for patients with ARDS (51 to 70%) and that the presence of ARDS was one factor independently associated with NIV failure and higher mortalities rates (50 to 70%). Recently, Zhan and colleagues [47] analyzed 40 patients with ARDS randomly allocated to receive either noninvasive ventilation or high-concentration oxygen therapy through a venturi mask. Noninvasive positive pressure ventilation decreased the respiratory rate and improved PaO_2_/FIO_2_ with time. The proportion of patients requiring intubation and invasive mechanical ventilation was significantly lower in the noninvasive ventilation group (one of 21 versus 7 of 19; *P* = 0.02). Therefore, noninvasive ventilation can be used as a first ventilatory support technique in selected patients with mild/moderate ARDS and a hemodynamic stable condition to avoid endotracheal intubation. A larger randomized trial, however, is required, with the need for intubation and mortality as the outcome of interest. A close-monitored initial trial of noninvasive ventilation should be considered in most mild/moderate ARDS patients, mainly the immunosuppressed ones with pulmonary infection in order to avoid intubation and invasive mechanical ventilation. However, early detection of NIV failure must be recognized, and a prompt intubation and mechanical ventilation must be provided in order to avoid complications.

## 6. Importance of Low Tidal Volume and Low Driving Pressure in ARDS

Protective ARDS mechanical ventilation strategies with tidal volumes equal to or less than 6 mL/Kg of predicted body weight have been traditionally associated with reduced mortality (when compared with 12 mL/Kg of predicted body weight) [48, 49]. A recent meta-analysis, however, scrutinized the specific role of various ventilatory strategies used in randomized trials on lung protection (like plateau-pressure limitation and higher PEEP use) and showed that tidal volume per se is not exactly the most important parameter to prioritize. The conclusion was that driving pressure (i.e., the difference between plateau pressure and PEEP during controlled mechanical ventilation) was the most important parameter to optimize at the bedside. Patients with high driving pressures (>16 cmH_2_O) may be at great danger even when using tidal volumes below 6 mL/kg, or even when presenting plateau-pressures below 30 cmH_2_O [50].

## 7. Importance of Static Pressure-Volume Curves of the Respiratory System to Set PEEP in ARDS

Amato and colleagues [51] demonstrated reduction in 28-day mortality in ARDS patients submitted to recruitment maneuver (CPAP 40 cmH_2_O) PEEP titrated by static Pressure × Volume (*P* × *V*) curve associated with low tidal volume (VT = 6 mL/kg), compared to those ventilated with high VT (12 mL/kg) and low PEEP strategy. It is hard to point out which component of such combined strategy was responsible for the benefits. Villar and colleagues [52] found congruent results in a similar protocol. Moreover, Ranieri and colleagues [53] demonstrated decreased lung inflammation with this protective ventilatory strategy. Although these results are encouraging, the physiologic background supporting the use of *P*-*V* curves to titrate PEEP lacks consistency nowadays. In many different situations, investigators have reported a large dissociation between closing pressures of the lung and the calculated value for the inflection point obtained from the inspiratory *P*-*V* curve. In general, patients with high values of inflection point tend to have a more severe disease, and this may explain the relative success of this strategy. Nevertheless, we will probably use better tools to titrate PEEP in the next few years. A more consistent use of the *P*-*V* curve has been demonstrated for the analysis of lung recruitability [54, 55].

## 8. Importance of Assisted Ventilation in ARDS Patients: Airway-Pressure Release, BIPAP, Pressure Support Ventilation, and NAVA**** Ventilation

Airway pressure release ventilation is a modified form of continuous positive airway ventilation (CPAP) described by Stock and Dows in 1987 that uses fairly high prolonged CPAP levels with short and intermittent releases of the airway pressure to low CPAP levels allowing ventilation and CO_2_ clearance. This mode of ventilatory support enhances oxygenation by augmenting alveolar recruitment and requires less sedation when used in ARDS patients compared to conventional mechanical ventilation [56, 57].

BiPAP ventilation combined with lung recruitment maneuvers can also be used in ARDS patients. Wang and colleagues compared this modality of ventilatory support with assist/controlled volume ventilation in a prospective, randomized trial of 28 ARDS patients showing a better PaO_2_/FIO_2_ ratio, pulmonary compliance, and a shorter duration of mechanical ventilation [58].

Pressure support ventilation (PSV) along with sufficient PEEP levels should be used as early as possible in ARDS patients to avoid respiratory muscle dystrophy and to decrease mechanical ventilation duration [32]. The reason for the improvement in oxygenation obtained with PSV in ARDS has been challenged in the recent years [59, 60]. The apparent improvement in recruitment seems to have been overstated and there is evidence that it is related to an increased perfusion of better ventilated lung areas, but not to decreased lung collapse. Growing concerns related to excessive tidal recruitment or excessive dyssynchrony during this mode of ventilation will have to be better addressed in the next years [61]. The advantages of using assist modes are to keep the respiratory muscles' activity, but sometimes it is difficult to synchronize the patients to the ventilators. Recently, neurally adjust ventilation (NAVA) was used in ARDS experimental models [62] and ARDS patients [63] demonstrating that the ventilation cycle and the magnitude of assist breath in NAVA matched the patients' breath pattern better than in PSV, NAVA improving patient-ventilator synchrony compared to PSV.

## 9. Importance of High Frequency Ventilation

High frequency oscillatory ventilation (HFOV) is an alternative mode of ventilatory support that can improve oxygenation by means of a higher mean airway pressure coupled with small tidal volumes generated by a piston pump oscillating at a frequency of 3–10 Hz and a higher respiratory rate. However, to date there are few studies involving a small number of patients comparing HFOV to conventional ventilation. A recent meta-analysis suggested a trend towards mortality benefit and more ventilator free days. However, the results of this analysis should be interpreted cautiously as the main study contributing to its results used high tidal volume in the control group rather than protective lung ventilation strategy [64].

## 10. Importance of Prone Position Ventilation

The use of the position change (supine to prone) leads to consistent improvement in arterial oxygenation in ARDS patients. Large randomized, controlled trials have consistently showed improvement in oxygenation without reduction in duration of mechanical ventilation or survival benefit. A recent meta-analyses suggest survival benefits in ARDS patients [65] or, more specifically, in a subgroup of patients with severe ARDS (PaO_2_/FIO_2_ < 100 mmHg) [66]. In our experience, the prone position can be an acceptable alternative to improve oxygenation in severe ARDS patients with arterial pulmonary hypertension and right ventricular dysfunction, which associated with the use of inhaled nitric oxide, can minimize intrathoracic pressures to facilitate right ventricular performance. The principles of a protective ventilation with proper PEEP titration and minimum driving pressures should also be pursued during prone positioning protocols.

## 11. Pulmonary Hypertension and Right**** Ventricular Dysfunction in ARDS Patients

Clinical studies suggested that elevated pulmonary artery systolic pressure in ARDS patients was associated with an adverse prognosis [67]. These data have been further supported by a more recent analysis of hemodynamic data from the ARDSNet Fluids and Catheter Therapy Trial (FACTT) [68]. The investigators assessed the transpulmonary gradient (TPG) (mean PA pressure-pulmonary capillary occlusion pressure (PCOP)) and the pulmonary vascular resistance index (PVRi) in a group of patients randomized to receive a pulmonary artery catheter to guide their ARDS management. Of note, all patients received a consistent protective ventilator strategy with target tidal volume ~6 mL/kg ideal body weight and plateau pressures maintained <30 cmH_2_O. The highest recorded daily value of TPG and PVRi was used for the analysis. In the population of 475 patients randomized to receive a pulmonary artery catheter for ARDS management, none of the baseline measures of cardiopulmonary dysfunction, including central venous pressure, PA systolic, or diastolic pressure, pulmonary capillary occlusion pressure (PAOP), or cardiac index distinguished survivors from nonsurvivors. In the pulmonary artery catheter population, 73% demonstrated an elevated transpulmonary gradient (TPG > 12). Patients with a TPG > 12 mmHg had a significantly greater mortality rate than patients with a TPG < 12 mmHg (30% versus 19%; *P* = 0.02). Patients with a persistently elevated TPG through day 7 of therapy had a significantly greater mortality than patients with an elevated TPG at day 0-1 which subsequently normalized. In multivariate analysis, pulmonary vascular dysfunction as represented by an elevated TPG and PVRi remained an independent predictor of an adverse outcome in the ARDS population. These data further support an important predictive role for pulmonary vascular disease in ARDS outcome [69]. In the largest published echocardiographic series of ARDS, 22% of patients receiving a consistent lung protective ventilation strategy (mean PEEP of 10 cmH_2_O and mean plateau pressure (Pplat) of 23 cmH_2_O) had evidence for acute cor pulmonale. In this population, 19% demonstrated evidence of a moderate-to-large patent foramen ovale [70]. The incidence of right to left shunting increased to 34% in patients with echocardiographic evidence of acute cor pulmonale.

## 12. Extracorporeal Membrane Oxygenation (ECMO) and Extracorporeal CO_**2**_ Removal in ARDS

Increase of oxygenation and CO_2_ removal by making the ARDS patients' blood pass throughout a membrane oxygenator outside the body is the principle of extracorporeal membrane oxygenation that can be applied venous-venous (good for oxygenation and CO_2_ removal), arterial-venous (good for CO_2_ removal), and venous-arterial (good for cardiovascular support). Early clinical trials of ECMO employed primarily an arterial-venous strategy with larger bore catheters for patients with intractable hypoxemia [71]. More modern investigations have used a safer venous-venous access approach [72, 73]. A recent UK prospective, randomized, clinical trial (CESAR) showed a survival advantage in the ECMO group (63% for ECMO versus 47% for controls). Nevertheless, the study was criticized as there was no standardized protocol management for the control group and some patients in the ECMO arm did not receive the proposed treatment [74]. The authors of CESAR trial also recommended transferring adult patients with severe but potentially reversible respiratory failure and a pH less than 7.20 on optimal conventional management, to a center with an ECMO-based management protocol to significantly improve survival without severe disability. The authors demonstrated that this strategy is also likely to be cost effective in settings with similar services to those in the United Kingdom [74]. Another recent approach for application of extracorporeal carbon dioxide removal new devices (ECMO-R) in ARDS patients is the demonstration that in severe ARDS even the low tidal volume ventilation with 6 mL/Kg of predicted body weight can cause tidal hyperdistension in the nondependent regions of the lungs accompanied by plateau airway pressures greater than 28 cmH_2_O and elevated plasma markers of inflammation. In this group application of ECMO-R could allow the authors to decrease the tidal volume to less than 6 mL/kg with a consequent plateau pressure less than 25 cmH_2_O that was associated with a lower radiographic index of lung injury and lower levels of lung-derived inflammatory cytokines. However, prognostic implication of this new ECMO-R devices application in clinical practice is still under investigation [75].

Pumpless interventional lung assist (iLA) is also used in patients with ARDS and is aimed at improving extracorporeal gas exchange with a membrane integrated in a passive arteriovenous shunt. iLA serves as an extracorporeal assist to support mechanical ventilation by enabling low tidal volume and a reduced inspiratory plateau pressure in extremely severe ARDS patients. Zimmermann and colleagues used iLA in 51 severe ARDS patients and observed a decrease in PaCO_2_ allowing the decrease in tidal volume and plateau pressure (ultraprotective ventilation) with a hospital mortality rate of 49% [76].

## 13. Combining Ventilatory Support Therapies in ARDS Patients

Some authors suggest the use of combined ventilatory strategies in patients with ARDS. Bingold and colleagues [77] successfully used superimposed high-frequency jet ventilation (SHFJV) in combination with continuous positive airway pressure/assisted spontaneous breathing (CPAP/ASB) in five patients with H1-N1-associated ARDS to improve oxygenation. Varpula and colleagues [78] demonstrated a significant improvement in oxygenation in 28 ARDS patients, when they compared APVR associated with prone ventilation to SIMV-Pressure control/pressure support group. APRV after 24 h appears to enhance improvement in oxygenation in response to prone positioning. Rival and colleagues [79] examined the effects of the prone position associated with a recruitment maneuver consisting of 45 cmH_2_O extended sigh in pressure control, in 16 ARDS patients. The combination of both ventilatory techniques led to the highest increase in PaO_2_/FIO_2_ ratio without significant clinical side effects. Lubnow and colleagues [80] examined the effects of 6 days of the combination of high-frequency oscillatory ventilation (HFOV) and extracorporeal carbon dioxide removal with the interventional lung assist (iLA) in 21 severe ARDS patients who failed conventional ventilation. They observed an increase in PaO_2_/FIO_2_ ratio and pH and a decrease in PaCO_2_. Weaning from HFOV/iLA was successful in 10 patients. The 30-day mortality rate was 43%, and hospital mortality rate was 57%.

In conclusion, combined ventilatory strategies can be applied in severe ARDS patients, but the best match among all the available ventilatory techniques is still a matter of debate.

## 14. Importance of Early Detection and**** Treatment of Infection/Inflammation Associated with ARDS

Pulmonary infection and sepsis are the most important triggering factors of ARDS. Pulmonary infection has been associated with a higher risk of ARDS progression in comparison to nonpulmonary infection in at risk populations [81]. A wide variety of organisms can invade the respiratory tract and trigger host innate and acquired immune system initiating the inflammatory cascade of ARDS, sepsis, and multiple organ failure [11]. It is particularly pertinent to investigate the etiology of pulmonary infection on the first day assessing a nasal swab for a respiratory virus detection (Influenza, adenovirus) lower respiratory tract secretion or a bronchoalveolar lavage fluid (BALF) for bacteria (especially multiresistant species), other viruses as herpes and cytomegalovirus, coronavirus, or metapneumonic virus [82]. Opportunistic agents such as Pneumocystis jiroveci must be investigated in immunosuppressed patients. Urinary screening for Legionella species is decisive, because if positive, specific therapy must be introduced [11]. The assessment of BALF on the first as well as on the third day of mechanical ventilation is of the utmost importance not only in terms of assessment of etiology of pulmonary infection but also of the assessment of proinflammatory mediators of ARDS (IL-1, IL6, IL8, IL 10, soluble tumor necrosis factor-alpha receptors (sTNFR), and soluble intercellular adhesion molecule-1) and mediators of ventilator-induced lung injury (that can also be obtained in the plasma) such as sTNFR, IL6, IL8, and IL-10, indicators of epithelial cell injury (soluble advanced glycation end-product receptors-sRAGE), and surfactant protein-D, components of the coagulation system (protein-C and plasminogen activator inhibitor 1) [11, 83]. Elevated levels of procollagen peptide III in lavage fluid from patients on day 3 of ARDS were independent risk factors for mortality [84].

Procalcitonin (PCT) and C-reactive protein (CRP) are progressively being used in critical care setting in order to diagnose pulmonary infection and sepsis and to guide the antibiotic therapy. Procalcitonin levels correlated with severe sepsis and bacteraemia [85]. A PCT-based algorithm guiding initiation and duration of antibiotic therapy in critical ill patients with suspected bacterial infections was associated with a 23% relative reduction in antibiotic exposure with no significant increase in mortality [86]. The persistence of an elevated serum CRP in critical ill patients with ARDS may alert the intensivist to a possible persistent infection or inflammatory process. At this moment, a new workup for infection and change in antibiotic therapy could help improve the patient's evolution. Early and quick administration of antibiotics in sepsis and septic shock as well as early goal resuscitative measures for septic shock or early goal-directed therapy decrease mortality in this high mortality critically ill conditions [87, 88].

We also suggest that preventive measures to avoid gastric aspiration (elevated decubitus, intermittent check for residual gastric content during diet infusion) and to avoid ventilation associated pneumonia (wash hands, elevated decubitus, special endotracheal tubes) should be implemented.

## 15. Resolution of ARDS

The resolution of pulmonary edema is central to recover from ALI as it entails regression of air space inflammation and restoration of a functioning alveolar-capillary membrane. Accordingly, elevated extravascular lung water measured using this technique early in the course of ALI/ARDS, particularly if indexed to predicted body weight, was associated with a poor prognosis [89].

## 16. Importance of Early Diagnosis, Evaluation, and Treatment of Multiple Organ Failure Associated with ARDS

**Figure 2:**
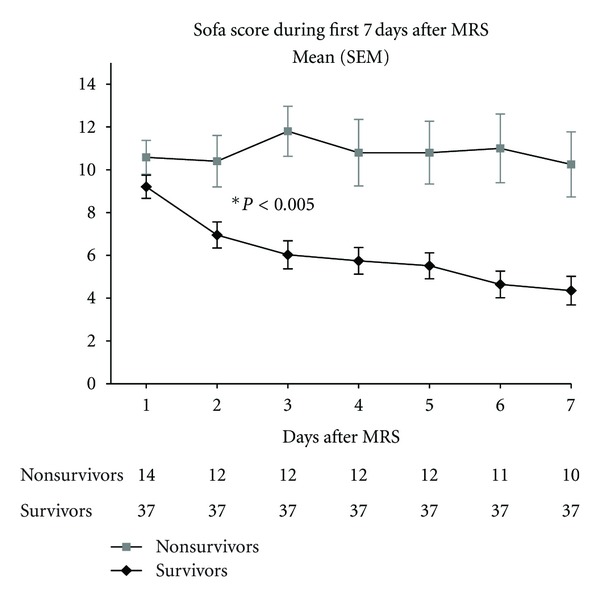
Sequential organ failure assessment (SOFA score) significantly decreases after the third day in ARDS survivors.

**Figure 3:**
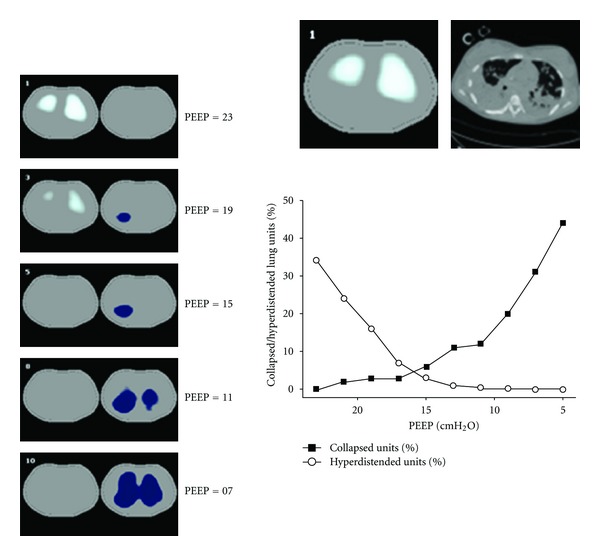
Peep titration with electrical impedance tomography in a patient with ARDS associated with H1N1-influenza virus infection. Legend: sequence of functional EIT images showing the progression of collapse along decremental PEEP levels (in blue, left panel) associated with progressive relief of overdistension (in white, left panel). Collapse was more prominent in the right lung. After analyzing the sequence of EIT images, the PEEP selected for this patient was 17 cmH_2_O, believed to represent the best compromise between collapse and overdistension. According to the ARDSNet PEEP/FIO_2_ table, this patient had been ventilated with a PEEP = 24 cmH_2_O in the previous 48 hours. The patient was weaned from ventilator 3 days later.

A study analyzing the evolution of ARDS patients showed that unknown-site infection (adjusted hazard ratio (HR) 3.08, 95% CI 1.37–6.90) and multiple site infection (adjusted HR 1.63, 95% CI 1.13–2.35) were associated with increased mortality [90]. In ARDS patients it is of considerable significance to evaluate the source of infection as well all organs and systems affected by the sepsis syndrome in order to map the organism (number of nonpulmonary organ failures), to calculate the prognostic indices (Acute Physiology and Chronic Health Evaluation (APACHE) and Simplified Acute Physiology Score (SAPS)) and to plan the multiorgan system approach to treat the disease. The higher the number of multiple organ failure associated with ARDS, the higher the hospital mortality. Trauma patients with ARDS are associated with lower mortality and oliguricrenal failure, while septic shock patients are associated with the highest hospital mortality rates, suggesting that during the first day of hospitalization these ARDS patients should be stratified and treated according to the severity of the syndrome and associated comorbidities [91]. In our case series of 51 patients with early severe ARDS the mean APACHE II score was 20.2 ± 6.2 (predicted mortality of 40%), median SOFA score (day 1) was 10 (7 to 12), median nonpulmonary organ failure was 2 (1 to 2), sepsis was present in 71% of our patients, and septic shock in 63%, vasopressors were used in 82.3% of our patients, and continuous renal replacement therapy was used in 56.8% of our patients. APACHE II and day 1 SOFA score were not associated with hospital mortality, but day 3 SOFA score was [33] (Figure 2) showing that a revaluation of the ARDS patients especially the ones with multiple organ failure and maintenance of SOFA score higher than 8 at day 3 has to be considered in order to evaluate hidden sources of infection or to change the antibiotics according to day 1 collected cultures.

## 17. Early Short-Term Paralysis in Severe ARDS Patients

In moderate-severe ARDS patients (PaO_2_/FIO_2_ < 150), a phase IV randomized controlled trial comparing cisatracurium to placebo for 48 hours showed an improved adjusted 90-day survival rate and increased ventilator-free in the cisatracurium group without a significant increase in muscle weakness. Short-term paralysis may facilitate patient-ventilator synchrony in the setting of lung protective ventilation. Short-term paralysis would eliminate patient triggering and expiratory muscle activity. In combination, these effects may serve to limit regional overdistention and cyclic alveolar collapse. Paralysis may also act to lower metabolism and overall ventilatory demand [92].

## 18. Importance of Other Pharmacological**** Therapies in ARDS Patients

Inhaled nitric oxide is an endogenous vasodilator that reduces *V*/*Q* mismatch and improves oxygenation by pulmonary vasodilation in alveolar units that are ventilated, reducing pulmonary vascular resistance in patients with ARDS. A Cochrane review of 14 clinical trials with 1303 patients showed only a transient improvement in oxygenation with no benefit regarding length of ICU or hospital stay, ventilator-free days or survival. An increased renal impairment was observed in the inhaled nitric oxide-treated group [93].

The effects of steroids in the late-stage fibrotic phase of ARDS (after 7 days of onset) were tested in a phase III study of the ARDS network. The study showed no mortality benefit in the treatment group, with a higher mortality in patients treated 14 days after onset [94]. Recently, Seam and colleagues tested the effects of methylprednisolone infusion in 55 early ARDS patients compared to placebo. They observed that methylprednisolone therapy was associated with greater improvement in lung injury score (*P* = 0.003), shorter duration of mechanical ventilation (*P* = 0.005), and lower intensive care unit mortality (*P* = 0.05) than in the control subjects. On days 3 and 7, methylprednisolone decreased interleukin-6 and increased protein-C levels (*P* < 0.001) compared with control subjects [95]. From the available evidence, low-dose steroids (1-2 mg/Kg/methylprednisolone) may be considered in patients with severe early ARDS. Nevertheless, it is not recommended to initiate corticosteroids beyond 14 days after the onset of ARDS.

Ketoconazole, lisofylline, sivelestat, N-acetylcysteine, and exogenous surfactant are not recommended as treatment for ARDS patients [12].

## 19. Importance of Fluid Management and**** Alveolar Fluid Clearance in ARDS Patients

Cumulative positive fluid balance is associated with worse clinical outcomes in patients with ARDS. A phase III study conducted by the ARDS network (the FACTT study) compared liberal versus conservative fluid strategy in patients with acute lung injury. They observed an improvement in oxygenation, lung injury score (LIS), and shortened duration of mechanical ventilation without any increase in other organ failure in the conservative group, despite no difference in hospital mortality [68].

Beta-agonists were investigated in multicenter, prospective, randomized trials in their aerosolized presentation (the ALTA study) and their intravenous presentation (the BALTI-2 study). Both studies showed no mortality benefit and Beta-agonists are not recommended as part of therapy for patients with ARDS [96].

## 20. Importance of Nutrition in ARDS Patients

The OMEGA study [97], a randomized, double-blind, placebo-controlled, multicenter trial analyzed 272 patients with early acute lung injury allocated to receive either twice-daily enteral supplementation of n-3 fatty acids, *γ*-linolenic acid, and antioxidants compared with an isocaloric control. Enteral nutrition, directed by a protocol, was delivered separately from the study supplement. The patients that received enteral supplementation had fewer ventilator-free days (14 versus 17.2, *P* = 0.02), more days with diarrhea (29 versus 21%; *P* = 0.001), and no difference in the adjusted 60-day mortality (25.1% versus 17.6%; *P* = 0.11). More recently, a randomized, open-label, multicenter trial, the Eden study [98], reported 1000 patients with acute lung injury, randomized to receive either trophic or full enteral feeding for the first 6 days. Initial trophic enteral feeding did not improve ventilator-free days, 60-day mortality, or infection complications but was associated with less gastrointestinal intolerance.

Finally, based on relevant literature articles and the authors' clinical experience, we suggest a goal-oriented management for critically ill patients with ARDS that can help improve clinicians' ability to care for these patients (as shown below).

**Algorithm for Goal-Oriented Management for Critically Ill Patients with ARDS**

*Correct ARDS Diagnosis.* Acute onset, increase respiratory rate, pulse oximeter desaturation and hypoxemia (PaO_2_/FIO_2_ < 300).

**Exposure to Traditional or Modified Risk Factors**

*Chest X-Ray Bilateral Pulmonary Infiltrates.*

- If possible, get a computer tomography (improved diagnosis accuracy, permits differential diagnoses, and helps to set recruitment maneuvers and adequate PEEP levels).
- Lung ultrasound, FDG-PET CT, electrical impedance tomography, and pressure-volume *P* × *V* curves can help assess the correct diagnosis and set protective mechanical ventilation.
- Get nasal swab and inferior respiratory tract secretion for infection diagnosis or a BAL (infection diagnosis and proinflammatory mediators and procollagen III measurements).
- Get hemocultures and blood for infection detection. Start resuscitative measurements for septic shock and start appropriate antibiotics.
- Assessment of prognostic indices (APACHE, SAPS) and sequential organ failure assessment (SOFA) score.

**Standardize Initial Mechanical Ventilation for Blood Gas Measurements**

Tidal volume: 6 mL/Kg predicted body weight, PEEP of 5 cmH_2_O, RR = 20.

**Classify ARDS Severity**

Mild: PaO_2_/FIO_2_ < 300, moderate: PaO_2_/FIO_2_ < 200, and severe: PaO_2_/FIO_2_ < 100.

- If possible, get a Doppler echocardiogram to assess left ventricular function, right ventricular function, systolic pulmonary artery pressure, and vena cava compressibility.
- Measure extravascular lung water, if available.
  - In cases of severe ARDS consider recruitment maneuvers and adequate PEEP titration.
  - In cases of severe ARDS with right ventricular dysfunction and pulmonary artery hypertension consider prone position and inhaled nitric oxide.
  - In cases of excessive CO_2_ retention: PaCO_2_ > 80 mmHg and pH < 7.2 consider intratracheal gas insufflation and extracorporeal CO_2_ removal.

## 21. Conclusions

- Early recognition of ARDS modified risk factors and avoidance of aggravating factors during hospital stay such as high tidal volume ventilation, multiple blood products transfusions, excessive fluid administration, ventilator associated pneumonia, and gastric aspiration prevention could help decrease its incidence.
- An early extensive clinical, laboratory, and imaging evaluation of “at risk patients” allows a correct diagnosis of ARDS, assessment of comorbidities, calculation of prognostic indices (APACHE, SAPS, SOFA), stratification of the severity of ARDS, and planning a careful treatment.
- Rapid administration of antibiotics and resuscitative measures in case of sepsis and septic shock associated with protective ventilatory strategies and early short-term paralysis associated with differential ventilatory techniques (recruitment maneuvers with adequate PEEP titration, prone position, and new ECMO techniques) in severe ARDS can help improve its prognosis.
- Revaluation of ARDS patients on the third day of evolution (SOFA, biomarkers, and response to infection therapy) allows changes in the initial treatment plans and can help decrease ARDS mortality.
- Fibroproliferative changes on high-resolution CT in ARDS can predict mortality and ventilator dependency.
